# Vaccination in the Light of the Royal British Commission

**Published:** 1899-01

**Authors:** M. R. Leverson

**Affiliations:** Fort Hamilton, L. I., N. Y.


### VACCINATION IN THE LIGHT OF THE ROYAL BRITISH COMMISSION.

M. R. Leverson, M. D., Fort Hamilton, L. I., N. Y.

(Continued from Dec. No., page 549.)

Qs. 10,096-99. See post 10,158.

Q. 10,100 (Dr. Collins). "Do I correctly understand your position to be that, viewing leprosy as an inoculable disease, one of the possible means of inoculation is the process of vaccination?" "Yes," and at Q. 10,128 Mr. Tebb goes further and calls it a probable cause. Pressed by Sir Wm. Savory as to whether he would go further than that he replied: "Not being a medical man, and not having had the kind of experience which would qualify me for giving a scientific answer, I think I have said as much as I am justified in saying."

Qs. 10,101-4. In answer to Dr. Collins he says that he regards the extension of vaccination as compatible with a diminution of leprosy, notwithstanding that leprosy is often invaccinated. Such diminution has actually occurred in Norway, where Dr. Hansen (identified with the discovery of the so-called leprosy bacillus) expresses that opinion. Mr. Tebb reads a letter from Dr. W. G. Armaur Hansen, dated Bergen, Norway, April 9th, 1889, in which he states that opinion.

Q. 10,107. Mr. Tebb gives facts from Trinidad and Barbados tending to show that leprosy is not contagious in the conventional use of that term.

Q. 10,138 goes next to Dr. Makuna's vaccination census or inquiry. "One of the most conspicuous examples of the conflict of medical opinion . . . . is exhibited in Dr. Makuna's *Transactions of the Vaccination Inquiry*, made by a committee of vaccine experts, in 1888." Several deaths from vaccination had occurred and it was desired by the advocates of vaccination to reinstate its credit. Hence the inquiry. The report was published, and has been analyzed by Mr. Thomas Baker. The report is called *Transactions of the Vaccination Inquiry*, and is edited by Montague D. Makuna, M. R. C. S. L. R. C. P. [The analysis was handed in, but the editor of this abstract has been unable to find a copy of it anywhere in any of the reports thus far published by the Royal Commission.]

There is no work readily accessible to either the public or the profession in which this report is to be found. It is to Mr. William Tebb that we are indebted for correcting this omission, by publishing the most important part of it as an appendix to his admirable work, *The Recrudescence of Leprosy*. Believing that it should be readily accessible, at least to the profession, it is here reproduced from that appendix:

About the end of the year 1882, or the beginning of 1883, Dr. Montague Makuna, Superintendent of the Fulham Small-pox Hospital, initiated an inquiry, by a medical commission, into the vaccination question. The meetings were presided over by Dr. C. R. Drysdale, Senior Physician of the Metropolitan Free Hospital.

"At the first meeting of the committee, held on the 15th February, 1883, Dr. Makuna, in explaining the objects of the proposed inquiry, referred to the opposition to vaccination; an opposition which has become intensified since vaccination has been made compulsory. A medical inquiry was, therefore, considered indispensable, and it was anticipated that the evidence disclosed in favor of vaccination would be so unanimous and conclusive as to effectually restore public confidence in the practice, and to put an end to all opposition. The Chairman, Dr. Drysdale, said he considered the proposed inquiry would be of great value to the profession and the public, and expressed a desire that the Local Government Board and other authorities should be requested to co-operate.

"The attention of the Local Government Board, the British Medical Association, the Epidemiological Society, the

Medical Officers of Health, the Royal College of Physicians, as well as Ambassadors, Consuls, etc., was especially called to the inquiry, and they were requested to contribute facts and information from all parts of the world.

"A circular was drawn up and approved by the council, and sent to 4,000 medical practitioners, a considerable portion of whom were public vaccinators, medical officers of health, and vaccine specialists. The circular elicited 384 answers, and the results were published in a pamphlet entitled, *Transactions of the Vaccination Inquiry*."

"An analysis of the answers (made by Mr. Thomas Baker, barrister-at-law), shows that the seven questions submitted have been answered by 384 medical men, of whom 102 are public vaccinators, vaccine specialists, medical officers of health, or officials.

"The following is the third, and one of the most important, submitted to this medical inquisition, viz.:

" 'What diseases have you, in your experience, known to be conveyed, or occasioned or intensified by vaccination?'

"To this question 13 give no answer, and 139 answer 'none,' but many qualify this reply by the words, 'in my own practice,' 'direct,' 'not serious,' 'personal,' etc."

It is also to be borne in mind that public vaccinators in England, commonly, do not see the vaccinated child after the eighth day; and many, perhaps most of the serious results of vaccination do not begin to show themselves until after that period.

"The lists of mischiefs (many fatal) includes the following, as recorded by 232 medical witnesses. [This enumeration has been checked by the Rev. Isaac Dixsey, F. S. S., and Mr. J. H. Lynn.]

| Mischief | No. of Witnesses |
|---|---|
| Abdominal phthisis, | 1 |
| Abscesses, | 11 |
| Angeioleucitis, | 2 |
| Arm diseases needing amputation, | 1 |
| Axillary bubo, | 1 |

| Mischief | No of Witnesses | Mischief | No. of Witnesses |
|---|---|---|---|
| Axillary gland, enlargment of, | 1 | Lichen, | 2 |
| Blindness, | 1 | Marasmus, | 1 |
| Blood poisoning (fatal), | 1 | Meningitis, | 2 |
| Boils, | 8 | Mesenteric disease, | 1 |
| Bronchitis, | 1 | Œdema, | 2 |
| Bullae, | 1 | Paralysis, | 1 |
| Cancer, | 1 | Phagedænic active, | 1 |
| Cellulitis, | 5 | Phlegmon, | 2 |
| Convulsions, | 4 | Pityriasis, | 1 |
| Diarrhœa, | 4 | Pneumonia, | 1 |
| "Died," | 1 | Prurigo, | 3 |
| Diseased bones, | 1 | Psoriasis, | 1 |
| Diseased joints, | 1 | Pyæmia, | 7 |
| Dyscrasia, | 1 | Pyrexia, | 1 |
| Ecthyma, | 1 | Rickets, | 1 |
| Eczema, | 60 | Scald head, | 1 |
| Eruptions, | 5 | Scarlatina, | 3 |
| Erysipelas, | 120 | Scrofula, | 9 |
| Erythema, | 22 | Septicæmia, | 1 |
| Gangrenosa, | 3 | Skin disease, | 21 |
| General Debility, | 1 | Struma, intensified, | 4 |
| Herpes, | 3 | Syphilis, | 43 |
| Impetigo, | 7 | Tuberculosis, | 1 |
| Inflammation, | 10 | Ulceration. | 6 |
| Latent diseases developed, | 2 | Varioloid, | 1 |

Qualified returns were also made of eczema by four witnesses; of erysipelas, by six; of nettlerash, by one, and of syphilis, by ten."

### Sitting of Wednesday, July 2d, 1890.

Q. 10,151. The Chairman objects to Mr. Baker's analysis as inaccurate. Taking cases of eczema at 61, some are of this kind; one says "possibly eczema;" another, "I have known eczema to spread from a vaccinated arm just as I have known it spread from any of the sources of irritation.* Another says, "eczema often alleged to be caused, but not on good evidence." As to syphilis one says, "I cannot quite as-

* Well, but why give this "source of irritation"?

sert that I have seen syphilis conveyed by vaccination, but I firmly believe I have seen such cases."

Q. 10,153. Mr. Tebb promises to have the figures checked.

Q. 10,155 (Chairman). "There are three cases in which scarlatina is said, according to the abstract, to have been caused or intensified by vaccination. There are only three cases in which scarlatina is said to have occured in reference to vaccination. One says, 'Scarlatina by the use of a lancet tainted with pus from a scarlatinal abscess in the practice of another medical man.' Another says, 'I had one case of scarlatina in which vaccination had been performed during the incubation period by another. Erysipelas set in, and the infant died of pyæmia and sloughing of the arm' (Mr. Tebb). That seems a strong case as against vaccination."

Q. 10,156. "The third is 'Three or four cases of eczema. In one it appeared very shortly after, reduced his strength, and died of scarlatina.' As far as I can understand, as far as the vaccination had anything to do with it, it was only the action of vaccination on a child not very strong, and therefore not so able to resist a disease contracted independently" (Mr. Tebb). "That is one of the points of our case."

Q. 10,158. At 10,096-9 Sir James Paget asked Mr. Tebb questions with regard to the spread of leprosy in other countries asserted by Mr. Tebb, to which he now gives answer, not having been prepared to do so at the previous session. He quotes Dr. H. F. D. Blanc, lecturer on dermatology, Tulane University of Louisiana (p. 63): "Leprosy is undoubtedly increasing in this city (New Orleans), slowly but steadily."

The Surgeon-General, of the Central Provinces of India, writing from Nagpur, August 10th, 1889: "If the census figures 1872-'81 are to be relied upon leprosy is increasing at an alarming rate. The number of persons recorded as lepers in the Central Provinces in the census returns of 1872 was 2,807; and the number recorded in 1881 was 6,443."

At a meeting of the Bombay Corporation the City Coroner stated that leprosy in Bombay was "vastly increasing." At the annual meeting of the Epidemiological Society, held in the Cavendish rooms, on June 12th, 1889, Surgeon-Major Pringle said that his opinion was "that leprosy was increasing in India."

At the same meeting Mr. R. Bradewell Carter said: "No doubt there was a preponderance of evidence that leprosy was on the increase in different parts of the world."

(Reported in the London *Globe*, June 13th, 1889.)

